# Magnetically Controlled Carbonate Nanocomposite with Ciprofloxacin for Biofilm Eradication

**DOI:** 10.3390/ijms22126187

**Published:** 2021-06-08

**Authors:** Viktoriya Rumyantceva, Valeriya Rumyantceva, Yulia Andreeva, Sofia Tsvetikova, Anton Radaev, Maria Vishnevskaya, Vladimir Vinogradov, Andrey S. Drozdov, Elena Koshel

**Affiliations:** 1 International Institute Solution Chemistry of Advanced Materials and Technologies, ITMO University, Lomonosova st., 9, 191002 St. Petersburg, Russia; viktoriya_rumyantceva@scamt-itmo.ru (V.R.); valeriya_rumyantceva@scamt-itmo.ru (V.R.); andreeva_9094@mail.ru (Y.A.); zvetikova@scamt-itmo.ru (S.T.); vinogradov@scamt-itmo.ru (V.V.); 2 Chromas Research Resource Center, St. Petersburg State University, 199034 St. Petersburg, Russia; anton.radaev@spbu.ru (A.R.); wishm@yandex.ru (M.V.); 3 Laboratory of Nanobiotechnology, Phystech School of Biological and Medical Physics, Moscow Institute of Physics and Technology, 9, 141701 Dolgoprudny, Moscow Region, Russia

**Keywords:** nanocomposite, magnetite, antibiotic, biofilm, antimicrobial effect

## Abstract

Biofilms are the reason for a vast majority of chronic inflammation cases and most acute inflammation. The treatment of biofilms still is a complicated task due to the low efficiency of drug delivery and high resistivity of the involved bacteria to harmful factors. Here we describe a magnetically controlled nanocomposite with a stimuli-responsive release profile based on calcium carbonate and magnetite with an encapsulated antibiotic (ciprofloxacin) that can be used to solve this problem. The material magnetic properties allowed targeted delivery, accumulation, and penetration of the composite in the biofilm, as well as the rapid triggered release of the entrapped antibiotic. Under the influence of an RF magnetic field with a frequency of 210 kHz, the composite underwent a phase transition from vaterite into calcite and promoted the release of ciprofloxacin. The effectiveness of the composite was tested against formed biofilms of *E. coli* and *S. aureus* and showed a 71% reduction in *E. coli* biofilm biomass and an 85% reduction in *S. aureus* biofilms. The efficiency of the composite with entrapped ciprofloxacin was higher than for the free antibiotic in the same concentration, up to 72%. The developed composite is a promising material for the treatment of biofilm-associated inflammations.

## 1. Introduction

The formation of biofilms on biotic and abiotic surfaces is a critical problem in different areas, from agriculture and industry to medicine. Since biofilms optimize the survival of most microorganisms, any surfaces can be subject to colonization, including the human body and medical devices. Implant infection of abiotic devices subjected to direct contact with a patient, such as catheters, prostheses, implants, is another severe and frequent medical complication [1,2,3]. Human tissues and organs are also subject to colonization by microorganisms, which provokes inflammation in diseases such as cystic fibrosis, otitis, and pericarditis [4].

Therapy of biofilm-related inflammation is a complex problem because bacteria inside a biofilm are 100–1000 times more resistant to conventional antibacterial agents effective against free-floating (plankton) bacterial cells. The low efficiency of biocides against biofilms is determined by their mechanical and chemical properties, which prevents deep penetration of the antibiotic. Another important issue is cells with reduced metabolism, known as ‘persisters’ [5]. Therefore, for effective antibiotic therapy, biofilms should be disintegrated beforehand. However, aggressive antibacterial compounds and rough mechanical action are unacceptable for use inside the body [6,7,8]. There is no practical and non-traumatic approach for addressing this problem so far. The most effective strategies against biofilms are still based on traumatic and expensive procedures for the removal of biofilms or replacement of the affected surfaces [4,8,9,10,11]. With the increase in implant-dependent operations and the biofilm-caused complications, the need to develop an effective therapy is continually increasing. The problems described above can be solved with a mechanical disintegration of the biofilm, followed by an intense release of antibiotics. For these tasks, biocide-conjugated nanoparticles (NPs) and nanocomposites have been intensively developed as an excellent opportunity for non-invasive therapy, as due to the small size and unique physicochemical properties, such systems can show unique and valuable properties for biomedical practice [12,13,14,15,16,17,18,19,20].

Taking into consideration the requirements for such systems, functional materials based on calcium carbonate can be considered as promising candidates for the creation of new types of functional materials due to their biocompatibility, synthetic availability, and good drug loading capacities [21,22,23,24,25]. Another interesting property of such systems originates from their crystalline structure and well-known capability to undergo a spontaneous transformation of its crystal phases from porous vaterite into dense calcite, which can be accompanied by an inevitable release of pre-immobilized agents [26]. Modified carbonate NPs can effectively prolong the activity of entrapped antibiotics and are shown to be active against planktonic forms of some microorganisms [27,28]. However, it is necessary to develop a target delivery system and controlled release in inflammatory foci for the potential application of carbonate-based drugs in therapy. This is important because the untargeted use of carbonate in high concentrations can lead to undesirable alkaline effects.

To solve the targeting problem, magnetically assisted delivery using NPs with superparamagnetic properties is currently proposed [18,29,30,31,32,33,34,35,36]. The most suitable material for this task is magnetite (Fe_3_O_4_), with proven efficacy for biocide-conjugated materials [12,29,37]. The popularity of magnetite nanoparticles (MNPs) in biomedicine is due to their high biocompatibility and magnetic controllability, allowing its use as a shuttle for biocide delivery directly within biofilm [38,39]. However, MNPs themselves have a low ability to conjugate biocide in such a way as to ensure its fully controlled release.

In the current study, we combined these two platforms to solve several biofilm control problems at once. This study describes a new class of antibiofilm agents: magnetically controlled carbonate nanocomposite with encapsulated model antibiotic—ciprofloxacin. Ciprofloxacin was chosen because of its high efficacy against microorganisms, and at the same time of low toxicity for mammals [40]. The system demonstrates two release profiles, either a slow sustained release or a burst release stimulated by a high-frequency magnetic field (210 kHz), which occurs due to accelerated crystal phase transformation and disintegration of composite particles. This process provides the optimal therapeutic action mode for the antibacterial agent at the site of inflammation.

The effectiveness of the developed nanocomposite has been proven in vitro in two bacterial models: Gram-negative *Escherichia coli* and Gram-positive *Staphylococcus aureus*. Our results showed that the nanocomposite was significantly more effective than the original antibiotic form in the same concentration. The efficiency improvement of the antibiotic in the nanocomposite was up to 72%. The results obtained could be used to develop practical approaches for antibiofilm therapy.

## 2. Results

### 2.1. Characterization of the Nanocomposite

**Figure 1:**
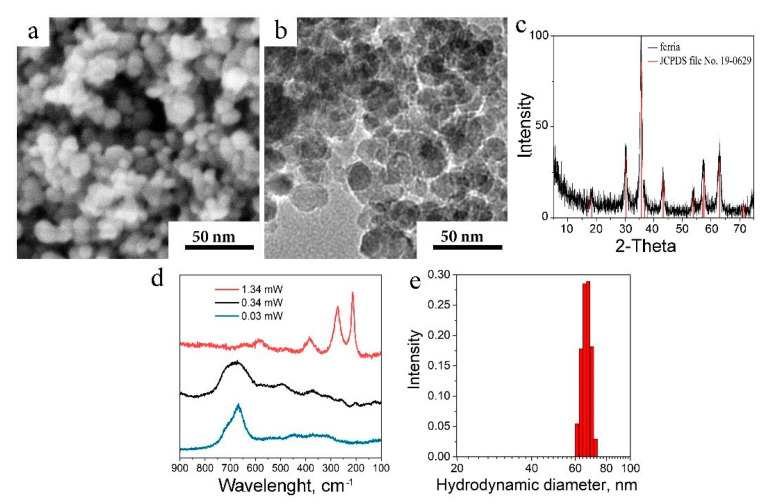
Characterization of the MNPs. (**a**) SEM image of MNPs; (**b**) TEM image of MNPs; (**c**) XRD pattern of MNPs compared to JCPDS file No. 19–0629; (**d**) Raman spectra of MNPs collected at various laser beam intensity; (**e**) DLS analysis of MNPs.

The nanocomposite was prepared by the co-entrapment of MNPs and the antibiotic ciprofloxacin into mesoporous vaterite-phase calcium carbonate particles. For this purpose, the highly stable hydrosol of pristine magnetite nanoparticles was used [41]. The hydrosol was prepared by ultrasonically assisted co-precipitation procedure and consisted of nanoparticles with a diameter of 10 nm and a narrow size distribution according to SEM (Figure 1a), TEM (Figure 1b), and X-ray diffraction (XRD) (Figure 1c) analysis. The XRD pattern of the MNPs corresponded to the magnetite crystal phase, and it was proved by Raman spectroscopy (Figure 1d). The spectra of MNPs demonstrated a typical transformation from magnetite to maghemite and hematite upon elevation of laser power from 0.03 to 1.34 mW. The MNPs zeta potential was valued + 34 mV at pH 7, which determined excellent colloidal stability of the material during synthesis of the final composite. Investigation of the as-prepared hydrosol by DLS showed that it consisted of aggregates with a mean hydrodynamic diameter of 63 nm (Figure 1e). MNPs demonstrated a superparamagnetic behavior with an almost zero coercivity, while magnetization values reached 76 emu/g at fields 7000 Oe (Figure S1).

**Figure 2:**
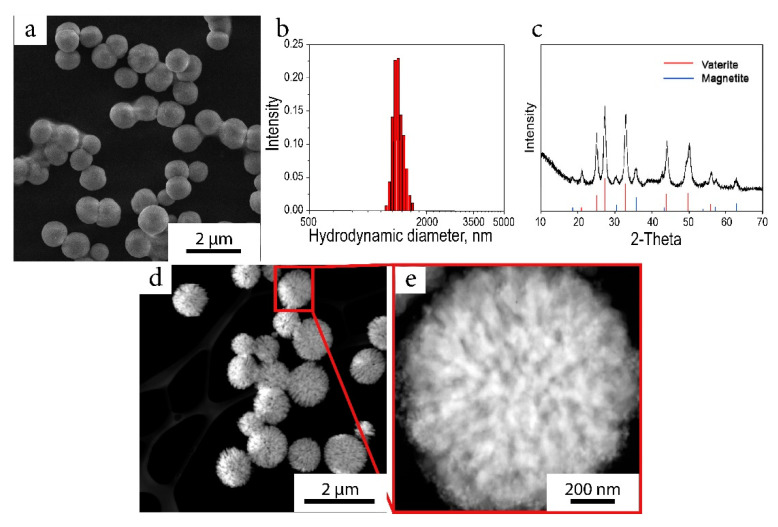
(**a**) SEM image of the magnetic composite particle; (**b**) the size distribution of hydrodynamic diameters in water solution; (**c**) XRD pattern of the composite particles; (**d**) STEM image of magnetic composite particles; (**e**) STEM image of the magnetic composite particles, in which the developed microstructure can be seen.

To produce the hybrid material, the MNP colloidal solution was mixed with ciprofloxacin and calcium chloride solutions with a subsequent sodium carbonate solution addition during constant stirring. As a result, calcium carbonate nucleation occurred on the surface of magnetite, and composite magnetite-carbonate microparticles were grown. During the synthesis, the positively charged amino groups of ciprofloxacin electrostatically interacted with the negatively charged carbonate groups resulted in the immobilization of the organic molecule within the porous composite. The resulted particles had a spherical morphology with a mean diameter of 1.3 µm with a narrow size distribution (Figure 2a,b); the XRD pattern of the material corresponded to a vaterite crystal phase (Figure 2c). STEM images of the particles demonstrated their loose porous structure with a developed architecture (Figure 2d,e). The material was mesoporous according to low-temperature nitrogen adsorption; the measured surface area calculated with the BET equation was 20 m^2^/g with a mean pore diameter of 3 nm according to a BJH model. The magnetic curves of the composite material were typical for superparamagnetic materials, but the magnetization values were low due to the presence of calcium carbonate and reached 11 emu/g at 7000 Oe (Figure S1). The amount of captured ciprofloxacin was evaluated by UV spectroscopy by measuring peaks at 274 and 320 nm in washing waters and was found to be 5% wt. for synthesized composite spheres.

**Figure 3:**
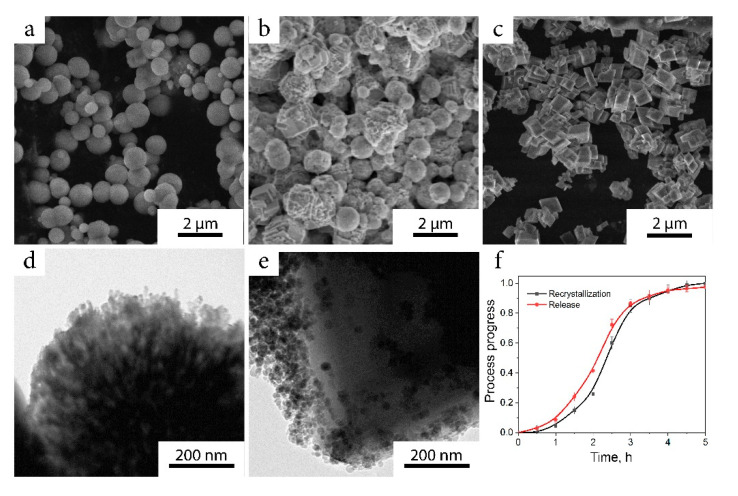
(**a**–**c**) Phase change of vaterite particles into calcite phase monitored by SEM; (**d**) TEM image of vaterite particle with even distribution of MNPs; (**e**) TEM image of calcite particle, MNPs are excluded to the surface; (**f**) release kinetics of ciprofloxacin compared to recrystallization dynamics.

In water media, the composite particles underwent a phase transition from vaterite into the calcite phase (Figure 3a–c). During this process, the morphology of the particles changed from spherical to cubic. Specific surface area reduced from 20 to 0.2 m^2^/g, and both MNPs and ciprofloxacin molecules were excluded from the particles into the media (Figure 3d,e). The release rate was in good correlation with the process of recrystallization and reached 97% after 5 h of incubation, following the recrystallization curve (Figure 3f).

**Figure 4:**
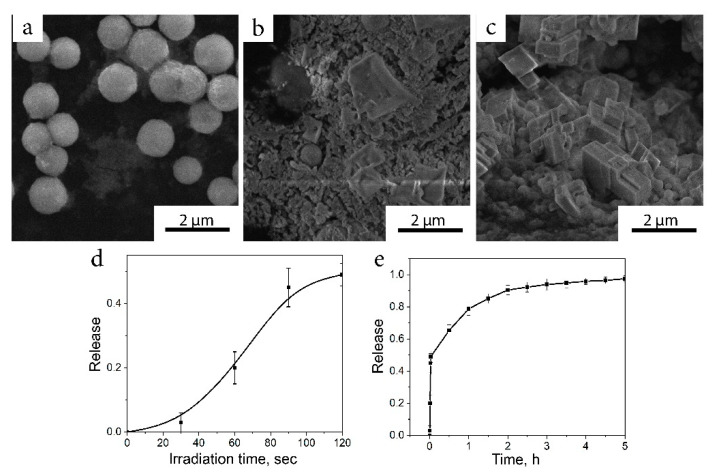
(**a**–**c**) Under the influence of the RF field, vaterite underwent blast-like destruction with a subsequent crystallization into the calcite phase; (**d**) under RF irradiation, burst release of ciprofloxacin occurred with (**e**) a subsequent slow release of the drug.

As an alternative to a passive release, the process can be triggered by an external stimulus by inducing a high-frequency (RF) magnetic field (210 kHz; 1 kA/m). With the field applied, the MNPs were heated up by Brownian and Néel relaxation up to 65 °C [42] to induce two synchronous processes: a porous vaterite transition of calcite to crystalline and local evaporation of water around MNPs. As a result, the destruction of the composite ceramic particles followed by a crystallization into larger crystallites and further release of the drug started (Figure 4a–c). Forty-nine percent of the total ciprofloxacin amount was released in a burst mode with a subsequent gradual release of the remaining antibiotic to a final 96% discharge of the content, which could cause a prolonged therapeutic effect (Figure 4d,e).

### 2.2. Nanocomposite Effectiveness Against the Formed Biofilms

**Figure 5:**
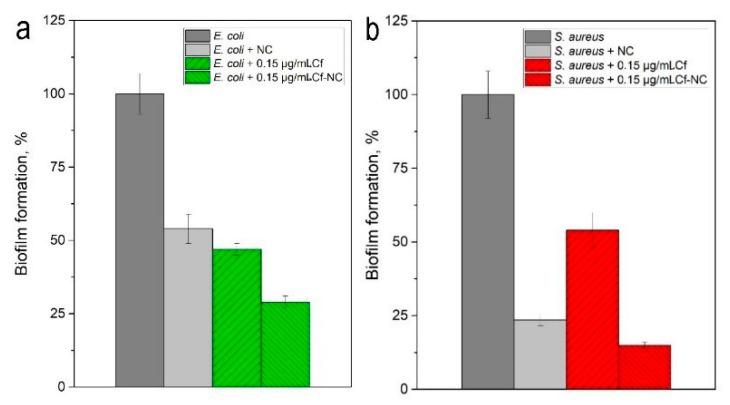
Efficacy of nanocomposite against the formed biofilms of (**a**) *E. coli* and (**b**) *S. aureus*. NC-treatment with a nanocomposite without antibiotic; Cf-treatment with ciprofloxacin; Cf-NC-treatment with ciprofloxacin immobilized in a nanocomposite.

The antibiofilm activity of the nanocomposite was tested on biofilms of model bacteria: *E. coli* (Gram-negative) and *S. aureus* (Gram-positive). For this purpose, biofilms were pre-formed on abiotic model surfaces: borosilicate glass and polystyrene. The composite was delivered and localized in the biofilm by a magnetic field using a neodymium magnet. Then, the composite was destroyed by exposure of the system into a high-frequency field (210 kHz and 1 kA/m) for 1 min, and the antibiotic was released. The biofilm biomass and the number of live cells within it were examined after 24 h of incubation (Table 1 and Figure 5). The amount of biomass was analyzed by the amount of binding dye (crystal violet) remaining after washing the biofilms. The higher the composite or antibiotic effectiveness, the smaller the amount of the dye bound to the biofilm, which was used to determine the quantitative characteristic of the latter.

**Figure 6:**
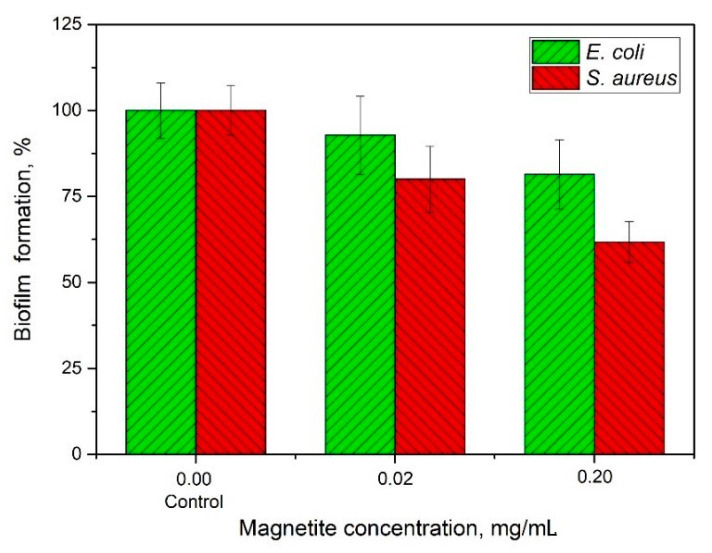
Efficacy of magnetite NPs against the formed biofilms: *E. coli* and *S. aureus*.

The antibiotic immobilized in a nanocomposite showed higher efficacy against formed biofilms than the antibiotic in its free form. Free antibiotic prevented biofilm growth by 53% in *E. coli* and by almost 46% in the case of *S. aureus*. At the same time, the nanocomposite with the antibiotic acted as follows: the biofilm proliferation reduction of *E. coli* and *S. aureus* by 71% and 85%, respectively. Thus, ciprofloxacin, initially less effective against *S. aureus* biofilm, was, on the contrary, more active. This may be due to the effect of the composite itself without the antibiotic. Under the same experimental conditions, the nanocomposite without the antibacterial agent reduced biofilm mass growth by 46% in *E. coli* and by 76.5% in *S. aureus*. Based on the data obtained, it is possible to judge the synergistic effect of nanocomposite and ciprofloxacin primarily for Gram-positive bacterial species—72% (*S. aureus*) vs. 38% (*E. coli*) (Table 1). This may be because Gram-positive bacteria are less protected from the surface and are more susceptible to the penetration of harmful ions formed, particularly under the action of magnetite [29]. First of all, the harmful effect of nanocomposite on biofilms is due to the magnetite contained in it. This was determined by studying the effect of magnetite on the biofilm outside the nanocomposite (Figure 6). MNPs reduced *E. coli* biofilm biomass by 18%, while *S. aureus* biofilm biomass was decreased by 34%. Thus, magnetite in the composition of the composite was used to determine its higher effectiveness against *S. aureus* biofilm.

**Figure 7:**
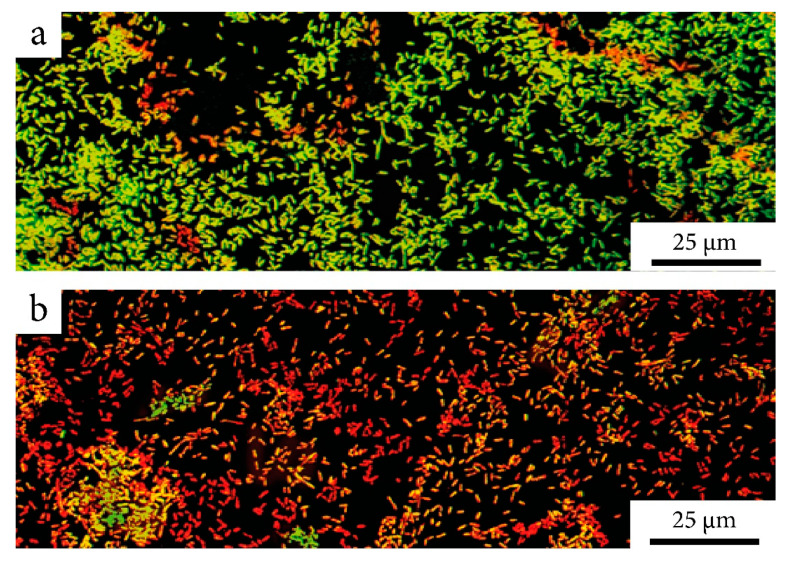
LIVE/DEAD staining of *E. coli* biofilms after treatment with ciprofloxacin 0.15 µg/mL: (**a**) in its original form; (**b**) immobilized into a nanocomposite. Confocal microscopy. Pseudocolors: red-dead cells and green-living cells.

In addition to assessing biofilm biomass after exposure to the composite, cell viability within the remaining biofilm was also examined. For this purpose, LIVE/DEAD staining followed by confocal microscopy was performed. Figure 7 shows the staining of *E. coli* biofilms after antibiotic treatment in their original form (Figure 7a) and as a part of composite (Figure 7b). As shown in the figure, the number of dead cells after exposure to a composite with an antibiotic was significantly higher than after antibiotic treatment in its free form. This indicates that even the biofilms that remained after exposure had weak viability.

## 3. Discussion

All modern strategies for treating biofilm-associated inflammation remain ineffective, and methods for the early detection, prevention, and elimination of biofilms are still lacking [43,44]. The conventional protocol is a combination of surgery and 6–12 weeks of systemic antimicrobial therapy [45]. This is harmful to various body systems, and the medical community was eager to get a less risky method for eradicating biofilms. Therefore, nanocomposite materials for invasive and non-invasive administration have been actively developed and used. One of the safe compounds is calcium carbonate, which is safely excreted from the human body. Obstacles to its use without specific initiators are the time required for the complex decomposition and the need to maintain additional working environment parameters. However, carbonate-based composite materials have found wide application, in particular, in antibiotic therapy. Thus, carbonate composites loaded with gentamicin have been used to treat bacterial bone infections [28]. Another study analyzed the prolonged effect of such composite systems on cancer cells [22] and the body’s immune system as a whole [24] in the form of potential components of anticancer and anti-inflammatory drugs. At the same time, it has been proven that, due to loading into a porous carbonate matrix, unstable compounds in an active state reach the target points of exposure to provide the expected effect, including an additional anti-infective effect.

At the same time, magnetic particles are not inferior in their popularity to use in various types of therapy. Embedding magnetite in the composite matrix solves two problems at once: the targeted delivery of an antibacterial agent and its controlled effect. At the same time, the release of active components from the matrix scaffold by external forced action occurs much faster than the effect of environmental changes in the pH through the bacteria’s vital activity [46]. In addition to this, the mechanical effect exerted under the influence of an external magnetic field on the disintegration of the bacterial biofilm makes it possible to reduce the amount of the antibacterial component loaded into the matrix [47]. Thus, the study of biocide-conjugated magnetic particles proved the better permeability of active agents into the biofilm thickness and its comparatively better eradication compared to a native antibiotic [47,48]. In this case, the initiation of the composite opening by a constant magnetic field was used. Revealing the efficiency of the system turned out to be a promising field for further study of its properties and modification options. The issue of controlled release under the influence of external controlled factors has not yet been considered in this context. Consequently, the not yet fully revealed potential of the complex was outlined.

It is known that a high-frequency magnetic field can be used for the controlled release of various active components from a composite matrix. For example, one study provides an example of using such a field to control the release of an anticancer agent [49]. In a study conducted on the MG63 human cell line, an additional antibiotic effect of the composite for the treatment of bone dystrophy in cancer was shown using the example of *S. aureus* [50], which is a prospect for development in this area. There is information on the use of high-frequency fields in the activation of an antiepileptic drug in the form of a specially designed flexible device [51]. It is known that in the case of magnetically induced release, there is an additional hyperthermic effect on damaged tissues [49,50], which causes an increase in their cytotoxic effect in cancer cells, in particular, apoptosis.

Concerning bacterial film cultures, there is ample evidence for the effects of a constant magnetic field on inflammation. In this case, the effect is not only on the sessile forms of bacteria but also on plankton ones, which are in dynamic equilibrium [29]. For the drug to attach to the surface of the biofilm, it is necessary to solve the problems of their affinity and delivery or to apply a modern concept: to use the ability to fully control internal processes through external ones. Mention of this can be found in some works, which talk about the use of a high-frequency magnetic field to release antibiotics to combat bacterial adhesion [50]. In addition, earlier studies were carried out for composites, which release the active component from the matrix under the action of ultrasound [52]. The efficiency was shown to be about 15%. However, even despite the prolonged effect, this was not enough for the treatment of biofilms rapidly developing on the surface.

The nanocomposite material presented in this paper provides an effective method for biofilm eradication. With magnetic targeting, the antibiotic in the inflammatory foci can be localized; due to the controlled opening of the composite particles under the influence of a 210 kHz magnetic field, an immediate antibiotic effect in high concentrations is possible. This gives the developed system a significant advantage in contrast to the systemic antibiotic intake associated with a higher dosage. Сiprofloxacin is а highly effective antibiotic compared to many others, but it is not sufficient to altogether remove bacteria in biofilms [53].

Our data also demonstrated the antibacterial effect of the nanocomposite without an antibiotic. This was primarily due to the presence of magnetite in the composition of the composite, which has a weak antibiofilm effect. The most significant effect is due to internal oxidative stress and cation release, which prevents cell proliferation due to DNA and protein disruption [18,29]. In addition, bacterial growth is blocked due to the accumulation of alkali ions on the film’s surface and the cells themselves. High alkalinity can inhibit bacterial growth and biofilm formation. An alkaline pH can affect the physicochemical properties of the bacterial surface and reduce the hydrophobicity of the bacterial surface, preventing bacterial adhesion [54,55].

Thus, the efficiency of the developed nanocomposite with immobilized ciprofloxacin was determined by (1) delivery to biofilm, (2) rapid antibiotic release due to which is obtained at a time in a high concentration, and (3) the synergistic antibacterial action of the antibiotic and composite. Due to these properties, the composite with antibiotics was 72% more effective than the antibiotic in its original form. Increased antibacterial activity of particles in *S. aureus* samples compared to *E. coli* could be an advantage to use against Gram-positive bacteria. The particles could also be loaded with other compounds for anti-inflammatory and antibacterial treatments.

The nanocomposite developed in this study is entirely biodegradable. MNPs are already widely used both as a drug and contrast agent for MRI [56], and vaterite is a native component of the human organism which undergoes biotransformation [57]. The magnetically-controlled vaterite matrix was considered to have low toxicity based on the previously obtained cytotoxicity and cellular uptake data [58].

Thus, the effectiveness of the presented nanocomposite for complex therapy of inflammatory diseases caused by the formation and development of biofilms on abiotic surfaces was proved. It is a promising groundwork for further in vivo studies for a comprehensive assessment of the possibility of using the developed complex in pharmacological and medical practice.

## 4. Materials and Methods

### 4.1. Materials

The components of an aqueous solution comprised of ammonia ≥27.5%, iron(II) chloride tetrahydrate ≥98.5%, iron(III) chloride hexahydrate ≥99%, anhydrous calcium chloride ≥99%, sodium carbonate ≥99%, ciprofloxacin ≥ 98.0% (HPLC), ethyl alcohol 99%, acetone for HPLC, crystal violet for biological stain, were obtained from Sigma–Aldrich (St. Louis, MO, USA). Deionized water 15 MOhm was produced with MilliQ Millipore Elix 3 system.

### 4.2. Bacterial Strain, Media, and Culture Conditions

This work used the *E. coli* ATCC 263-116 and *S. aureus* 209P strains. Cells were grown in LB medium (Lennox, Sigma–Aldrich).

### 4.3. MNPs Synthesis

MNPs synthesis was based on the original method described in [59]. Following this method, magnetite hydrosol was obtained by the co-condensation of iron(II) and (III) chlorides by adding an aqueous solution of ammonia followed by washing to neutral pH ultrasonic treatment. The resulting hydrosol had a mass fraction of solids of 2% wt.

### 4.4. Nanocomposite Synthesis

One milliliter of the stable hydrosol of MNPs was mixed with 100 µL of the 1% ciprofloxacin solution and a 100 µL of 0.33 M calcium chloride solution. Under intense stirring, 100 µL of 0.33 M sodium carbonate solution was added to the mixture and stirred at 500 rpm for 5 min. After that, the synthesized composite was filtered through a syringe filter with an average pore size of 1 μm to remove large aggregates, which was then lyophilized and stored under sterile conditions at +4 °C. Before use, the composite was dispersed in sterile deionized water to a final concentration of 1%.

### 4.5. Antibiotic Release

The release of antibiotics was examined spectrophotometrically using Agilent Cary 8454 UV-Vis (Malaysia). In a quartz cuvette, 5 mg of the composite was mixed with 1 mL of sterile deionized water and incubated at 37 °C for five hours in the spectrophotometer. The release of the drug was monitored by UV spectra measurements at 277 and 320 nm.

### 4.6. Characterization

Zeta potential and hydrodynamic radius were measured by the method of dynamic light scattering using a Photocor Compact-Z analyzer (Moscow, Russia). To analyze the samples using transmission electron microscopy (TEM), the crushed xerogel was deposited on a carbon-coated copper substrate, and then the sample was examined using an FEI TECNAI G2 F20 electron microscope (FEI Company, Hillsboro, OR, USA). For analysis using scanning electron microscopy (SEM), the samples were dried in a vacuum for 1 h and examined using a Tescan VEGA 3 scanning electron microscope with complete set of UniVac (Tescan Orsay Holding, Brno, Czech). The crystalline phase and the crystallinity of the samples were measured by X-ray diffraction (XRD) with a Bruker D8 Advance diffractometer (Brucker, Billerica, MA, USA) using Cu Kα radiation (λ = 1.54 Å). Samples were scanned at 2θ at a rate of 0.5 degrees per minute. The specific surface area, average pore diameter, and total pore volume were determined by nitrogen adsorption-desorption using a Quantachrome Nova 1200 (Quantachrome Instruments, Boynton Beach, FL, USA). Before the analysis, the samples were degassed for 2 h at 373 K. The surface area was calculated using the Brunauer–Emmett–Teller (BET) equation. The average pore diameter was calculated by the Barret–Joyner–Halenda (BJH) method. Raman spectra were recorded using a 633 nm He-Ne laser line using a Horiba Jobin Yvon MicroRaman 300 instrument (Horiba Jobin Yvon, Bensheim, Germany) with a 50× Olympus lens and a diffraction grating with 1800 grooves/mm. For all measurements, the hole was 500 μm, and the gap was 100 μm. The spectral analysis and antibiotic release kinetics were performed with an Agilent Cary 8454 UV-Vis spectrometer (Agilent, Santa Clara, CA, USA) coupled with a Temperature Controller unit. The radiofrequency (RF) field was generated by commercially available Geekcreit 5V-12V ZVS (Geekcreit, Shenzhen, China) induction heating power supply module with a water-cooled coil; the coil parameters were: number of turns—10 and length—0.03 m. The inductor was powered by a TPR3003T 3C Triple channel DC (Shenzhen Atten Electronics Co, Shenzhen, China) regulated power supply. The field frequency was 210 kHz with an amplitude of 1 kA/m.

### 4.7. Studies of the Nanocomposite Effectiveness Against Formed Biofilms

Studies of the nanocomposite effectiveness were carried out in an in vitro model using two abiotic surfaces: borosilicate glass and polystyrene. Biofilms were prepared by preincubating cell suspensions (10^6^ cells/mL) in a growth medium on borosilicate glass slides and 96-well immunological plates at 37 °C. After washing out the plankton cells, fresh growth medium and (1) nanocomposite with ciprofloxacin, (2) nanocomposite without ciprofloxacin, or (3) ciprofloxacin in its original form were added to the samples to compare the effectiveness. Ciprofloxacin was used at concentrations of 0.15 µg/mL and 2.5 µg/mL for *E. coli* and *S. aureus*, respectively. The nanocomposite with antibiotic was used in such an amount that the ciprofloxacin concentration corresponded to the concentrations mentioned above. A composite without antibiotics was used in the same concentration minus the mass of ciprofloxacin.

After the addition of nanocomposite alone or with the antibiotic, they were immersed in biofilms under a constant magnetic field; then, the nanocomposite was destroyed by a magnetic field of 210 kHz and 1 kA/m’ for 1 min. After 24 h incubation at 37 °C, the plates were prepared for quantitative analysis, and the slides—for microscopic analysis.

### 4.8. Studies of MNPs Against Formed Biofilms

The experiements were carried out in the same way as in Section 4.7. The magnetite solution added to the biofilm samples was used at concentrations of 0.02 and 0.20 mg/mL to reveal the dynamics of their growth suppression. The final concentration of magnetite was taken similar to that used in the nanocomposite.

### 4.9. Quantitative Evaluation of Biofilm

To quantify biofilms, they were fixed with 4% paraformaldehyde and stained with 0.1% crystal violet. The dye bound to the biofilm was later extracted with a mixture of ethyl alcohol and acetone (3:1). The concentration of the released dye was measured spectrophotometrically by a microplate reader Tecan Infinite^®^ F50 at a wavelength of 600 nm [60].

### 4.10. Microscopic Analysis of Biofilm

For microscopic analysis of biofilms to evaluate the bacterial cell viability, the slides were fixed with 4% paraformaldehyde and treated with BacLight Live/Dead stain (Ex: 488 nm/Em: 500 and 635 nm; Thermo Fisher Scientific) based on the manufacturer’s protocol. The microscopic analysis of the biofilm was carried out in a Leica TCS SP5 confocal scanning laser microscope using a Leica 63× oil immersion objective.

### 4.11. Statistical Analysis

All experiments were performed at least three independent sets, and data were represented as mean ± SD. All graphical evaluations were made using GraphPad Prism 7.0 (GraphPad Software Inc., La Jolla, CA). Analysis of variance (ANOVA) was used to evaluate the significant differences, and *p* < 0.05 was considered significant.

## Figures and Tables

**Table 1:** Efficacy of nanocomposite against the formed biofilms of *E. coli* and *S. aureus*.

Compound	Biofilm Mass after Treatment (М ± SD)		The Efficiency of CF in NC Compared To the Original Form of CF, %
	CF ^1^ in Its Original Form (Free)	CF in NC ^2^	
***E. coli***			
Without compound	0.951 ± 0.07		-
NC	0.516 ± 0.05		-
NC with 0.15 µg/mL CF	0.443 ± 0.02	0.275 ± 0.02	38
***S. aureus***			
Without compound	1.575 ± 0.08		-
NC	0.370 ± 0.02		-
NC with 2.5 µg/mL CF	0.853 ± 0.06	0.239 ± 0.01	72

^1^ Ciprofloxacin. ^2^ Nanocomposite.

## Data Availability

Not applicable.

## Supplementary Materials

The following are available online at https://www.mdpi.com/article/10.3390/ijms22126187/s1.

## References

[1] Arciola C.R., Campoccia D., Ehrlich G.D., Montanaro L., Donelli G. (2015). Biofilm-based implant infections in orthopaedics. Biofilm-Based Healthcare-Associated Infections.

[2] Arciola C.R., Campoccia D., Montanaro L. (2018). Implant infections: Adhesion, biofilm formation and immune evasion. Nat. Rev. Microbiol..

[3] Montanaro L., Speziale P., Campoccia D., Ravaioli S., Cangini I., Pietrocola G., Giannini S., Arciola C.R. (2011). Scenery *of Staphylococcus* implant infections in orthopedics. Future Microbiol..

[4] Bouza E., Garcia-Garrote F., Cercenado E., Marin M., Diaz M.S. (1999). *Pseudomonas aeruginosa*: A Survey of Resistance in 136 Hospitals in Spain. Antimicrob. Agents Chemother..

[5] Prax M., Bertram R. (2014). Metabolic aspects of bacterial persisters. Front. Cell. Infect. Microbiol..

[6] Römling U., Kjelleberg S., Normark S., Nyman L., Uhlin B.E., Åkerlund B. (2014). Microbial biofilm formation: A need to act. J. Intern. Med..

[7] Ribet D., Cossart P. (2015). How bacterial pathogens colonize their hosts and invade deeper tissues. Microbes Infect..

[8] Percival S.L., Suleman L., Vuotto C., Donelli G. (2015). Healthcare-associated infections, medical devices and biofilms: Risk, tolerance and control. J. Med. Microbiol..

[9] Høiby N., Bjarnsholt T., Moser C., Bassi G.L., Coenye T., Donelli G., Hall-Stoodley L., Holá V., Imbert C., Kirketerp-Møller K. (2015). ESCMID guideline for the diagnosis and treatment of biofilm infections 2014. Clin. Microbiol. Infect..

[10] Mermel L.A., Allon M., Bouza E., Craven D.E., Flynn P., O’Grady N.P., Raad I.I., Rijnders B.J.A., Sherertz R.J., Warren D.K. (2009). Clinical Practice Guidelines for the Diagnosis and Management of Intravascular Catheter-Related Infection: 2009 Update by the Infectious Diseases Society of America. Clin. Infect. Dis..

[11] Lebeaux D., Ghigo J.M., Beloin C. (2014). Biofilm-related infections: Bridging the gap between clinical management and fundamental aspects of recalcitrance toward antibiotics. Microbiol. Mol. Biol. Rev..

[12] Chifiriuc M.C., Grumezescu A.M., Andronescu E., Ficai A., Cotar A.I., Grumezescu V., Bezirtzoglou E., Lazar V., Radulescu R. (2013). Water dispersible magnetite nanoparticles influence the efficacy of antibiotics against planktonic and biofilm embedded Enterococcus faecalis cells. Anaerobe.

[13] Niemirowicz K., Durnaś B., Tokajuk G., Głuszek K., Wilczewska A.Z., Misztalewska I., Mystkowska J., Michalak G., Sodo A., Wątek M. (2016). Magnetic nanoparticles as a drug delivery system that enhance fungicidal activity of polyene antibiotics. Nanomedicine.

[14] Cotar A.I., Grumezescu A.M., Huang K.-S., Chifiriuc C.M., Radulescu R. (2013). Magnetite nanoparticles influence the efficacy of antibiotics against biofilm embedded *Staphylococcus aureus* cells. Biointerface Res. Appl. Chem..

[15] Cotar A.I., Grumezescu A.M., Andronescu E., Voicu G., Ficai A., Ou K.-L., Huang K.-S., Chifiriuc M.C. (2013). Nanotechnological solution for improving the antibiotic efficiency against biofilms developed by gram-negative bacterial strains. Lett. Appl. NanoBioSci..

[16] Fakhardo A.F., Anastasova E.I., Gabdullina S.R., Solovyeva A.S., Saparova V.B., Chrishtop V.V., Koshevaya E.D., Krivoshapkina E.F., Krivoshapkin P.V., Kiselev G.O. (2019). Toxicity Patterns of Clinically Relevant Metal Oxide Nanoparticles. ACS Appl. Bio Mater..

[17] Dukhinova M.S., Prilepskii A.Y., Shtil A.A., Vinogradov V.V. (2019). Metal oxide nanoparticles in therapeutic regulation of macrophage functions. Nanomaterials.

[18] Shkodenko L.A., Kassirov I.S., Koshel E.I. (2020). Metal Oxide Nanoparticles Against Bacterial Biofilms: Perspectives and Limitations. Microorganisms.

[19] Paramonova A.P., Kiselev G.O., Fakhardo A.F., Krivoshapkin P.V., Krivoshapkina E.F. (2019). Synthesis of upconversion zirconia nanoparticles for bioimaging. IOP Conf. Ser. Mater. Sci. Eng..

[20] Andreeva Y.I., Drozdov A.S., Solovyeva A.S., Fakhardo A.F., Vinogradov V.V. (2020). Polyelectrolyte-based magnetic photonic crystals with anticoagulant activity. Mater. Today Chem..

[21] Yan X., Li J., Möhwald H. (2012). Templating assembly of multifunctional hybrid colloidal spheres. Adv. Mater..

[22] Dizaj S.M., Barzegar-Jalali M., Zarrintan M.H., Adibkia K., Lotfipour F. (2015). Calcium carbonate nanoparticles as cancer drug delivery system. Expert Opin. Drug Deliv..

[23] Koroleva L.F., Dobrinskaya M.N., Kamantsev I.S. (2015). Doped nanocrystalline calcium carbonate-phosphate—A biomaterial for bone repair and strengtheining by drug delivery. Diagn. Resour. Mech. Mater. Struct..

[24] Ueno Y., Futagawa H., Takagi Y., Ueno A., Mizushima Y. (2005). Drug-incorporating calcium carbonate nanoparticles for a new delivery system. J. Control. Release.

[25] Trofimov A.D., Ivanova A.A., Zyuzin M.V., Timin A.S. (2018). Porous inorganic carriers based on silica, calcium carbonate and calcium phosphate for controlled/modulated drug delivery: Fresh outlook and future perspectives. Pharmaceutics.

[26] Chang R., Kim S., Lee S., Choi S., Kim M., Park Y. (2017). Calcium carbonate precipitation for CO_2_ storage and utilization: A review of the carbonate crystallization and polymorphism. Front. Energy Res..

[27] Ataee R.A., Derakhshanpour J., Mehrabi Tavana A., Eydi A. (2011). Antibacterial effect of calcium carbonate nanoparticles on *Agrobacterium tumefaciens*. J. Mil. Med..

[28] Dizaj S.M., Lotfipour F., Barzegar-Jalali M., Zarrintan M.-S., Adibkia K. (2016). Physicochemical characterization and antimicrobial evaluation of gentamicin-loaded CaCO_3_ nanoparticles prepared via microemulsion method. J. Drug Deliv. Sci. Technol..

[29] Rumyantceva V.I., Rumyantceva V.I., Koshel E.I., Vinogradov V.V. (2019). Biocide-conjugated magnetite nanoparticles as an advanced platform for biofilm treatment. Ther Deliv..

[30] Grumezescu A.M., Cristescu R., Chifiriuc M.C., Dorcioman G., Socol G., Mihailescu I.N., Mihaiescu D.E., Ficai A., Vasile O.R., Enculescu M. (2015). Fabrication of magnetite-based core–shell coated nanoparticles with antibacterial properties. Biofabrication.

[31] Laurent S., Saei A.A., Behzadi S., Panahifar A., Mahmoudi M. (2014). Superparamagnetic iron oxide nanoparticles for delivery of therapeutic agents: Opportunities and challenges. Expert Opin. Drug Deliv..

[32] Neuberger T., Schöpf B., Hofmann H., Hofmann M., Von Rechenberg B. (2005). Superparamagnetic nanoparticles for biomedical applications: Possibilities and limitations of a new drug delivery system. J. Magn. Magn. Mater..

[33] Scialabba C., Licciardi M., Mauro N., Rocco F., Ceruti M., Giammona G. (2014). Inulin-based polymer coated SPIONs as potential drug delivery systems for targeted cancer therapy. Eur. J. Pharm. Biopharm..

[34] Prilepskii A.Y., Fakhardo A.F., Drozdov A.S., Vinogradov V.V., Dudanov I.P., Shtil A.A., Bel’tyukov P.P., Shibeko A.M., Koltsova E.M., Nechipurenko D.Y. (2018). Urokinase-conjugated magnetite nanoparticles as a promising drug delivery system for targeted thrombolysis: Synthesis and preclinical evaluation. ACS Appl. Mater. Interfaces.

[35] Prilepskii A.Y., Schekina A.N., Vinogradov V.V. (2019). Magnetically controlled protein nanocontainers as a drug depot for the hemostatic agent. Nanotechnol. Sci. Appl..

[36] Anastasova E.I., Prilepskii A.Y., Fakhardo A.F., Drozdov A.S., Vinogradov V.V. (2018). Magnetite nanocontainers: Toward injectable highly magnetic materials for targeted drug delivery. ACS Appl. Mater. Interfaces.

[37] Inozemtseva O.A., German S.V., Navolokin N.A., Bucharskaya A.B., Maslyakova G.N., Gorin D.A., Nikolelis D.P., Nikoleli G.-P. (2018). Encapsulated Magnetite Nanoparticles: Preparation and Application as Multifunctional Tool for Drug Delivery Systems. Nanotechnology and Biosensors.

[38] Taylor E.N., Kummer K.M., Durmus N.G., Leuba K., Tarquinio K.M., Webster T.J. (2012). Superparamagnetic iron oxide nanoparticles (SPION) for the treatment of antibiotic-resistant biofilms. Small.

[39] Ranmadugala D., Ebrahiminezhad A., Manley-Harris M., Ghasemi Y., Berenjian A. (2017). The effect of iron oxide nanoparticles on *Bacillus subtilis* biofilm, growth and viability. Process Biochem..

[40] Davis R., Markham A., Balfour J.A. (1996). Ciprofloxacin. Drugs.

[41] Drozdov A.S., Ivanovski V., Avnir D., Vinogradov V.V. (2016). A universal magnetic ferrofluid: Nanomagnetite stable hydrosol with no added dispersants and at neutral pH. J. Colloid Interface Sci..

[42] Andreeva Y.I., Drozdov A.S., Avnir D., Vinogradov V.V. (2018). Enzymatic nanocomposites with radio frequency field-modulated activity. ACS Biomater. Sci. Eng..

[43] Gilbert P., Das J., Foley I. (1997). Biofilm susceptibility to antimicrobials. Adv. Dent. Res..

[44] Hall-Stoodley L., Costerton J.W., Stoodley P. (2004). Bacterial biofilms: From the natural environment to infectious diseases. Nat. Rev. Microbiol..

[45] Trampuz A., Zimmerli W. (2006). Diagnosis and treatment of infections associated with fracture-fixation devices. Injury.

[46] Serov N.S., Darmoroz D.D., Lokteva A.V., Chernyshov I.Y., Koshel E.I., Vinogradov V.V. (2020). One-pot synthesis of template-free hollow anisotropic CaCO_3_ structures: Towards inorganic shape-mimicking drug delivery systems. Chem. Commun..

[47] Geilich B.M., Gelfat I., Sridhar S., van de Ven A.L., Webster T.J. (2017). Superparamagnetic iron oxide-encapsulating polymersome nanocarriers for biofilm eradication. Biomaterials..

[48] Bandara H.M.H.N., Nguyen D., Mogarala S., Osinski M., Smyth H.D.C. (2015). Magnetic fields suppress *Pseudomonas aeruginosa* biofilms and enhance ciprofloxacin activity. Biofouling..

[49] Hardiansyah A., Huang L.-Y., Yang M.-C., Liu T.-Y., Tsai S.-C., Yang C.-Y., Kuo C.-Y., Chan T.-Y., Zou H.-M., Lian W.-N. (2014). Magnetic liposomes for colorectal cancer cells therapy by high-frequency magnetic field treatment. Nanoscale Res. Lett..

[50] Bigham A., Aghajanian A.H., Behzadzadeh S., Sokhani Z., Shojaei S., Kaviani Y., Hassanzadeh-Tabrizi S.A. (2019). Nanostructured magnetic Mg_2_SiO_4_-CoFe_2_O_4_ composite scaffold with multiple capabilities for bone tissue regeneration. Mater. Sci. Eng. C.

[51] Huang W.C., Hu S.-H., Liu K.-H., Chen S.-Y., Liu D.-M. (2009). A flexible drug delivery chip for the magnetically-controlled release of anti-epileptic drugs. J. Control. Release..

[52] Norris P., Noble M., Francolini I., Vinogradov A.M., Stewart P.S., Ratner B.D., Costerton J.W., Stoodley P. (2005). Ultrasonically controlled release of ciprofloxacin from self-assembled coatings on poly (2-hydroxyethyl methacrylate) hydrogels for *Pseudomonas aeruginosa* biofilm prevention. Antimicrob. Agents Chemother..

[53] Ghanwate N.A. (2012). Biofilm eradication studies on uropathogenic *E. coli* using ciprofloxacin and nitrofurantoin. Int. J. Pharm. Biomed. Res..

[54] Padan E., Bibi E., Ito M., Krulwich T.A. (2005). Alkaline pH homeostasis in bacteria: New insights. Biochim. Biophys. Acta Biomembr..

[55] Nostro A., Cellini L., Giulio M.D., D′Arrigo M., Marino A., Blanco A.R., Favaloro A., Cutroneo G., Bisignano G. (2012). Effect of alkaline pH on staphylococcal biofilm formation. Apmis.

[56] Revia R.A., Zhang M. (2016). Magnetite nanoparticles for cancer diagnosis, treatment, and treatment monitoring: Recent advances. Mater. Today.

[57] Tolba E., Müller W.E.G., Abd El-Hady B.M., Neufurth M., Wurm F., Wang S., Schröder H.C., Wang X. (2016). High biocompatibility and improved osteogenic potential of amorphous calcium carbonate/vaterite. J. Mater. Chem. B.

[58] Serov N.S., Prilepskii A.Y., Sokolov A., Vinogradov V.V. (2019). Synthesis of Plasmin-Loaded Fe_3_O_4_@CaCO_3_ Nanoparticles: Towards Next-Generation Thrombolytic Drugs. ChemNanoMat.

[59] Shapovalova O.E., Drozdov A.S., Brushkova E.A., Morozov M.I., Vinogradov V.V. (2020). Room-temperature fabrication of magnetite-boehmite sol-gel composites for heavy metal ions removal. Arab. J. Chem..

[60] Merritt J.H., Kadouri D.E., O’Toole G.A. (2005). Growing and Analyzing Static Biofilms. Curr. Protoc. Microbiol..

