# Supplementary material for: Magnetically Controlled Carbonate Nanocomposite with Ciprofloxacin for Biofilm Eradication
Source: Int J Mol Sci. 2021 Jun 8;22(12):6187. doi: 10.3390/ijms22126187 (PMC8229197; doi:10.3390/ijms22126187)
Supplement: Supplementary file 1 [file ijms-22-06187-s001.zip › ijms-1236347-supplementary.pdf]

Supporting materials for

Magnetically controlled carbonate nanocomposite with ciprofloxacin for biofilms eradication

Viktoriya Rumyantceva [1], Valeriya Rumyantceva [1], Yulia Andreeva [1], Sofia Tsvetikova [1], Anton Radaev [2], Maria Vishnevskaya [2], Vladimir Vinogradov [1], Andrey S. Drozdov [1,3]* and Elena Koshel [1,*]

1 International Institute Solution Chemistry of Advanced Materials and Technologies, ITMO University, Lomonosova st., 9, 191002, St. Petersburg, Russia; viktoriya_rumyantceva@scamt-itmo.ru (Vi.R.); valeriya_rumyantceva@scamt-itmo.ru (Va.R.); andreeva_9094@mail.ru (Y.A.); zvetikova@scamt-itmo.ru (S.T.); vinogradov@scamt-itmo.ru (V.V.).

2 St. Petersburg State University, 199034, St. Petersburg, Russia; anton.radaev@spbu.ru (A.R.); wishm@yandex.ru (M.V.)

3 Moscow Institute of Physics and Technology, Laboratory of Nanobiotechnology, Moscow Region, Russia; drozdov.as@mipt.ru (A.S.D.).

* Correspondence: koshel@scamt-itmo.ru; Tel.: 8-(931) 579-29-93

*

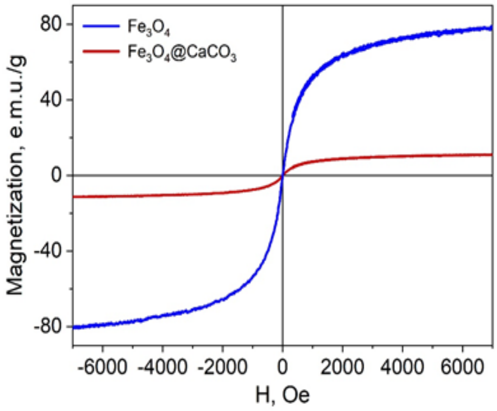

Figure S1: Magnetization curves of the magnetite NPs and composite material.
